# 2434. Healthcare-associated Infections in the Era of COVID-19 Pandemic: An Experience from a Tertiary Care Hospital in Long Island, New York

**DOI:** 10.1093/ofid/ofad500.2053

**Published:** 2023-11-27

**Authors:** Monirul I Sajib, Tamasin Adams, Susan V Donelan, Roderick Go, Miguel A Saldivar

**Affiliations:** Stony Brook University Hospital, Stony Brook, New York; Stony Brook University Hospital, Stony Brook, New York; Stony Brook Medicine, Stony Brook, NY; Stony Brook University Hospital, Stony Brook, New York; Stony Brook University Hospital, Stony Brook, New York

## Abstract

**Background:**

Healthcare-associated infections (HAIs) such as central line-associated bloodstream infections (CLABSI), catheter-associated urinary tract infections (CAUTI), surgical site infections (SSI) and ventilator-associated events (VAE) are associated with increased patient morbidity, mortality, and healthcare related cost. One could expect a decrease in the incidence of HAIs during the COVID-19 pandemic due to the enhanced infection control measures and increased awareness among the healthcare workers. The aim of our study is to assess the prevalence of HAIs during the 2 years of COVID-19 pandemic compared to 2 years prior to the pandemic at Stony Brook University Hospital, located in Long Island, NY.

**Methods:**

Standardized infection ratio (SIR) and percentile data for CLABSI, CAUTI, SSI and VAE were collected from the National Healthcare Safety Network website for hospitalized patients between January 2018 to December 2019 and between July 2020 and June 2022. Data between January 2020 to June 2020 was not available due to voluntary reporting at the beginning of pandemic. Superficial SSI cases were excluded. Data on organisms involved in CLABSI during this period were also collected.

**Results:**

During January 2018 to December 2019, SIR (percentile) for CLABSI, CAUTI, SSI and VAE are 0.623 (38), 0.455 (31), 1.129 (72) and 0.941 (44) respectively, whereas during July 2020 to June 2022, SIR (percentile) are 0.558 (34), 0.854 (63), 0.870 (56) and 1.316 (53) respectively, with *p*-value of 0.6149, 0.0007, 0.2471, and 0.0035 and relative risk of 0.896, 1.877, 0.771, and 1.399 respectively. In addition, during January 2018 to December 2019, *S. aureus* (8), coagulase-negative *Staphylococcus* (10), *Streptococcus* (2), *Enterococcus* (12), gram-negative rods (21) and *Candida* spp (11) were associated with CLABSI, versus *S. aureus* (5), coagulase-negative *Staphylococcus* (2), *Streptococcus* (2), *Enterococcus* (14), gram-negative rods (19), and *Candida* spp (13) during July 2020 to June 2022.

Comparison of HAIs during 2 years prior to the COVID-19 pandemic to 2 years of pandemic.

**Figure:**
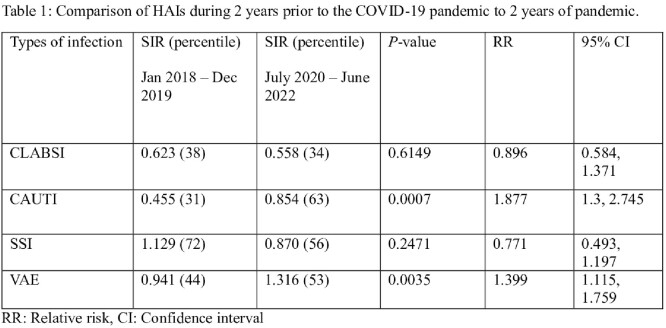

Organisms associated with CLABSI during 2 years prior to the COVID-19 pandemic compared to 2 years of pandemic

**Figure:**
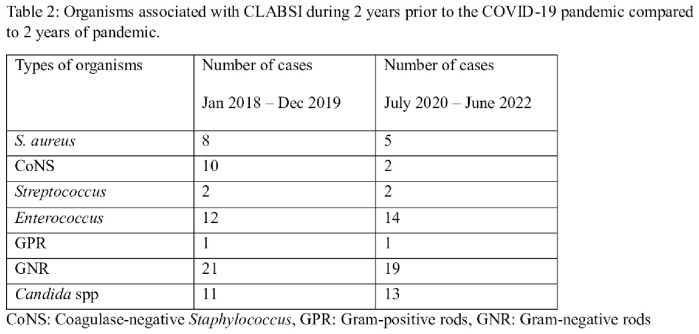

**Conclusion:**

We did not observe a significant reduction in CLABSI and SSI cases during the 2 years of COVID-19 pandemic compared to pre-pandemic phase; however, a significant rise in CAUTI and VAE cases were seen which we suspect is due to the increased urinary catheter and ventilator utilization during the COVID-19 pandemic.

**Disclosures:**

**Roderick Go, DO**, Aptose Biosciences: Stocks/Bonds|Bristol Meyers Squibb: Stocks/Bonds|Cytodyn Inc.: Stocks/Bonds|Scynexis: Grant/Research Support

